# Supplementary material for: Tuning excited state electronic structure and charge transport in covalent organic frameworks for enhanced photocatalytic performance
Source: Nat Commun. 2023 Feb 27;14:1106. doi: 10.1038/s41467-023-36710-x (PMC9970987; doi:10.1038/s41467-023-36710-x)
Supplement: Supplementary file 1 — Supplementary Information [file 41467_2023_36710_MOESM1_ESM.pdf]

Supplementary Information

Tuning Excited State Electronic Structure and Charge Transport in Covalent Organic Frameworks for Enhanced Photocatalytic Performance

Zhongshan Chen,[1] Jingyi Wang,[1] Mengjie Hao,[1] Yinghui Xie,[1] Xiaolu Liu,[1] Hui Yang,*[,1] Geoffrey I. N. Waterhouse,[2] Xiangke Wang,*[,1] and Shengqian Ma*[,3]

[1] College of Environmental Science and Engineering, North China Electric Power University, Beijing 102206, P.R. China;

[2] School of Chemical Sciences, The University of Auckland, Auckland 1142, New Zealand;

[3] Department of Chemistry, University of North Texas, Denton, Texas 76201, United States

Email: h.yang@ncepu.edu.cn (H.Y.); xkwang@ncepu.edu.cn (X.W.); sqma@usf.edu (S.M.)

Supplementary Table 1. Fractional atomic coordinates for the eclipsed AA-stacking unit cell of COF-1.

Space group *P*1, a = 30.30 Å, b = 30.54 Å, c = 3.49 Å, $\alpha = \beta$ =90°, and γ =117.98° Pawley refinement R_p = 1.58%, R_{wp} = 2.27%							
Atom	x (Å)	y (Å)	z (Å)	Atom	x (Å)	y (Å)	z (Å)
C1	0.59486	0.36269	0.5	C71	0.04356	0.49168	0.5
N2	0.39265	0.59063	0.5	C72	0.4609	0.01496	0.5
N3	0.42964	0.57547	0.5	C73	0.44836	0.96087	0.5
C4	0.41688	0.5241	0.5	O74	0.98385	0.39979	0.5
C5	0.45733	0.50951	0.5	O75	0.42223	0.0282	0.5
C6	0.44799	0.46177	0.5	C76	0.02801	0.3944	0.5
C7	0.49018	0.44852	0.5	C77	0.0139	0.33984	0.5
O8	0.37293	0.49305	0.5	C78	0.37273	0.98813	0.5
O9	0.3981	0.42394	0.5	C79	0.33574	0.0084	0.5
C10	0.39125	0.37454	0.5	O80	0.73784	0.2934	0.5
C11	0.33593	0.33847	0.5	H81	0.55371	0.33387	0.5
C12	0.36734	0.65443	0.5	H82	0.4697	0.60355	0.5
C13	0.38278	0.70521	0.5	H83	0.48558	0.42443	0.23796
C14	0.34767	0.72288	0.5	H84	0.48744	0.42583	0.7669
C15	0.29635	0.6891	0.5	H85	0.40933	0.36835	0.76693
C16	0.28054	0.63758	0.5	H86	0.40934	0.36836	0.2331
C17	0.31627	0.62086	0.5	H87	0.33009	0.30002	0.58305
C18	0.22768	0.5996	0.5	H88	0.31996	0.33699	0.20145
C19	0.36431	0.77651	0.5	H89	0.31606	0.35124	0.71549
N20	0.19148	0.61072	0.5	H90	0.42434	0.7328	0.5
N21	0.4112	0.80845	0.5	H91	0.30394	0.57939	0.5
N22	0.14193	0.57304	0.5	H92	0.21746	0.5586	0.5
N23	0.42573	0.85922	0.5	H93	0.3356	0.79072	0.5
C24	0.10119	0.58433	0.5	H94	0.13373	0.5336	0.5
C25	0.04903	0.54321	0.5	H95	0.39698	0.87204	0.5
O26	0.10996	0.6276	0.5	H96	0.93606	0.51222	0.23796
C27	0.47731	0.89536	0.5	H97	0.93578	0.5104	0.7669
C28	0.49121	0.94898	0.5	H98	0.5758	0.05743	0.23796
O29	0.50909	0.88152	0.5	H99	0.57437	0.05794	0.7669
C30	0.00885	0.55116	0.5	H100	0.94901	0.58724	0.76693
C31	0.95644	0.50832	0.5	H101	0.94901	0.58722	0.2331
C32	0.5391	0.98504	0.5	H102	1.00036	0.67643	0.20374
C33	0.55164	0.03913	0.5	H103	1.01667	0.68035	0.72167
O34	0.01615	0.60021	0.5	H104	0.9519	0.66481	0.57459
O35	0.57777	0.9718	0.5	H105	0.63316	0.03553	0.76693

C36	0.97199	0.6056	0.5	H106	0.63314	0.03553	0.2331
C37	0.9861	0.66016	0.5	H107	0.67524	0.98858	0.19229
C38	0.62727	0.01187	0.5	H108	0.64643	0.95335	0.63902
C39	0.66426	0.9916	0.5	H109	0.69898	1.01762	0.66869
O40	0.26216	0.7066	0.5	H110	0.27715	0.74163	0.66659
C41	0.40514	0.63731	0.5	H111	0.44629	0.66613	0.5
N42	0.60735	0.40937	0.5	H112	0.5303	0.39645	0.5
N43	0.57036	0.42453	0.5	H113	0.51442	0.57557	0.23796
C44	0.58312	0.4759	0.5	H114	0.51256	0.57417	0.7669
C45	0.54267	0.49049	0.5	H115	0.59067	0.63165	0.76693
C46	0.55201	0.53823	0.5	H116	0.59066	0.63164	0.2331
C47	0.50982	0.55148	0.5	H117	0.67589	0.67816	0.20145
O48	0.62707	0.50695	0.5	H118	0.68602	0.64118	0.58305
O49	0.6019	0.57606	0.5	H119	0.67199	0.69241	0.71549
C50	0.60875	0.62546	0.5	H120	0.57566	0.2672	0.5
C51	0.66407	0.66153	0.5	H121	0.69606	0.42061	0.5
C52	0.63266	0.34557	0.5	H122	0.78254	0.4414	0.5
C53	0.61722	0.29479	0.5	H123	0.6644	0.20928	0.5
C54	0.65233	0.27712	0.5	H124	0.86627	0.4664	0.5
C55	0.70365	0.3109	0.5	H125	0.60302	0.12796	0.5
C56	0.71946	0.36242	0.5	H126	0.06394	0.48778	0.23796
C57	0.68373	0.37914	0.5	H127	0.06422	0.4896	0.7669
C58	0.77232	0.4004	0.5	H128	0.4242	0.94257	0.23796
C59	0.63569	0.22349	0.5	H129	0.42563	0.94206	0.7669
N60	0.80852	0.38928	0.5	H130	0.05099	0.41276	0.76693
N61	0.5888	0.19155	0.5	H131	0.05099	0.41278	0.2331
N62	0.85807	0.42696	0.5	H132	0.03738	0.33262	0.72167
N63	0.57427	0.14078	0.5	H133	0.02108	0.32871	0.20374
C64	0.89881	0.41567	0.5	H134	-0.02738	0.31708	0.57459
C65	0.95097	0.45679	0.5	H135	0.36684	0.96447	0.76693
O66	0.89004	0.3724	0.5	H136	0.36686	0.96447	0.2331
C67	0.52269	0.10464	0.5	H137	0.29933	-0.01968	0.63902
C68	0.50879	0.05102	0.5	H138	0.32814	0.01555	0.19229
O69	0.49091	0.11848	0.5	H139	0.35188	0.04459	0.66869
C70	0.99115	0.44884	0.5	H140	0.77129	0.31898	0.66659

Supplementary Table 2. Fractional atomic coordinates for the eclipsed AA-stacking unit cell of COF-2.

Space group $P1$, a = 30.10 Å, b = 31.23 Å, c = 3.48 Å, $\alpha = \beta$ = 90°, and γ = 119.33° Pawley refinement R_p = 4.54%, R_{wp} = 7.21%							
Atom	x (Å)	y (Å)	z (Å)	Atom	x (Å)	y (Å)	z (Å)
C1	0.58883	0.36159	0.5	C72	0.044	0.49235	0.5
N2	0.39668	0.59203	0.5	C73	0.4583	0.01106	0.5
N3	0.43298	0.57636	0.5	C74	0.44828	0.95868	0.5
C4	0.41802	0.5253	0.5	O75	0.98222	0.40114	0.5
C5	0.45779	0.51014	0.5	O76	0.41713	0.02053	0.5
C6	0.4464	0.46265	0.5	C77	0.02688	0.39638	0.5
C7	0.48797	0.44879	0.5	C78	0.01181	0.34249	0.5
O8	0.37278	0.49499	0.5	C79	0.3677	0.97847	0.5
O9	0.39494	0.42566	0.5	C80	0.32789	0.99474	0.5
C10	0.38597	0.37649	0.5	O81	0.7316	0.29356	0.5
C11	0.32916	0.34134	0.5	O82	0.55759	0.26078	0.5
C12	0.37411	0.65623	0.5	H83	0.54649	0.33345	0.5
C13	0.39108	0.70684	0.5	H84	0.4742	0.60376	0.5
C14	0.35542	0.72393	0.5	H85	0.48234	0.42483	0.23755
C15	0.30294	0.68981	0.5	H86	0.48426	0.42619	0.76732
C16	0.28613	0.63896	0.5	H87	0.40376	0.37004	0.76735
C17	0.32189	0.62287	0.5	H88	0.40377	0.37005	0.23268
C18	0.23203	0.60092	0.5	H89	0.32167	0.30304	0.58212
C19	0.37101	0.77679	0.5	H90	0.31312	0.34021	0.20126
N20	0.19552	0.61141	0.5	H91	0.30988	0.35431	0.71662
N21	0.41815	0.81092	0.5	H92	0.30871	0.58186	0.5
N22	0.1448	0.5737	0.5	H93	0.22106	0.56042	0.5
N23	0.43042	0.8603	0.5	H94	0.34087	0.78885	0.5
C24	0.10369	0.5843	0.5	H95	0.13593	0.53476	0.5
C25	0.05028	0.54316	0.5	H96	0.39964	0.87015	0.5
O26	0.11319	0.62701	0.5	H97	0.93541	0.51123	0.23755
C27	0.48225	0.89878	0.5	H98	0.9351	0.50943	0.76732
C28	0.49372	0.95084	0.5	H99	0.57593	0.06066	0.23755
O29	0.51625	0.88819	0.5	H100	0.57441	0.06102	0.76732
C30	0.00969	0.55048	0.5	H101	0.94959	0.58524	0.76735
C31	0.956	0.50765	0.5	H102	0.94959	0.58523	0.23268
C32	0.5417	0.98894	0.5	H103	1.00244	0.67361	0.20236
C33	0.55172	0.04132	0.5	H104	1.01975	0.67784	0.71963
O34	0.01778	0.59886	0.5	H105	0.95372	0.66168	0.57801
O35	0.58287	0.97947	0.5	H106	0.63711	0.04447	0.76735

C36	0.97312	0.60362	0.5	H107	0.63709	0.04448	0.23268
C37	0.98819	0.65751	0.5	H108	0.68449	0.00421	0.19233
C38	0.6323	0.02153	0.5	H109	0.65534	-0.03293	0.63231
C39	0.67211	0.00526	0.5	H110	0.70649	0.03224	0.67536
O40	0.2684	0.70644	0.5	H111	0.28404	0.7412	0.66575
O41	0.44241	0.73922	0.5	H112	0.45098	0.76896	0.71065
C42	0.41117	0.63841	0.5	H113	0.45351	0.66655	0.5
N43	0.60332	0.40797	0.5	H114	0.5258	0.39624	0.5
N44	0.56702	0.42364	0.5	H115	0.51766	0.57517	0.23755
C45	0.58198	0.4747	0.5	H116	0.51574	0.57381	0.76732
C46	0.54221	0.48986	0.5	H117	0.59624	0.62996	0.76735
C47	0.5536	0.53735	0.5	H118	0.59623	0.62995	0.23268
C48	0.51203	0.55121	0.5	H119	0.68334	0.67518	0.20126
O49	0.62722	0.50501	0.5	H120	0.69189	0.638	0.58212
O50	0.60506	0.57434	0.5	H121	0.6801	0.68927	0.71662
C51	0.61403	0.62351	0.5	H122	0.69129	0.41814	0.5
C52	0.67084	0.65866	0.5	H123	0.77894	0.43958	0.5
C53	0.62589	0.34377	0.5	H124	0.65913	0.21115	0.5
C54	0.60892	0.29316	0.5	H125	0.86407	0.46524	0.5
C55	0.64458	0.27607	0.5	H126	0.60036	0.12985	0.5
C56	0.69706	0.31019	0.5	H127	0.06459	0.48877	0.23755
C57	0.71387	0.36104	0.5	H128	0.0649	0.49057	0.76732
C58	0.67811	0.37713	0.5	H129	0.42407	0.93934	0.23755
C59	0.76797	0.39908	0.5	H130	0.42559	0.93898	0.76732
C60	0.62899	0.22321	0.5	H131	0.05041	0.41476	0.76735
N61	0.80448	0.38859	0.5	H132	0.05041	0.41477	0.23268
N62	0.58185	0.18908	0.5	H133	0.03581	0.33575	0.71963
N63	0.8552	0.4263	0.5	H134	0.0185	0.33152	0.20236
N64	0.56958	0.1397	0.5	H135	-0.03022	0.3196	0.57801
C65	0.89631	0.4157	0.5	H136	0.36289	0.95553	0.76735
C66	0.94972	0.45684	0.5	H137	0.36291	0.95552	0.23268
O67	0.88681	0.37299	0.5	H138	0.29112	0.96472	0.63231
C68	0.51775	0.10122	0.5	H139	0.32028	1.00187	0.19233
C69	0.50628	0.04916	0.5	H140	0.34228	1.02989	0.67536
O70	0.48375	0.11181	0.5	H141	0.76588	0.31933	0.66575
C71	0.99031	0.44952	0.5	H142	0.53846	0.27303	0.71065

Supplementary Table 3. Fractional atomic coordinates for the eclipsed AA-stacking unit cell of COF-3.

Space group $P3$, $a = b = 30.51$ Å, $c = 3.92$ Å, $\alpha = \beta = 90°$, and $\gamma = 120°$ Pawley refinement $R_p = 3.96\%$, $R_{wp} = 6.22\%$							
Atom	x (Å)	y (Å)	z (Å)	Atom	x (Å)	y (Å)	z (Å)
C1	0.59744	0.36759	0.13147	C24	0.67911	0.64264	1.03916
N2	0.39856	0.58948	0.6565	C25	0.63057	0.35242	0.04298
N3	0.43573	0.57577	0.60343	C26	0.61211	0.29772	-0.00232
C4	0.42749	0.52856	0.68518	H27	0.55556	0.33805	0.11103
C5	0.46714	0.51478	0.62193	H28	0.47293	0.60351	0.49258
C6	0.45477	0.46994	0.48066	H29	0.4831	0.41673	0.29564
C7	0.49432	0.45476	0.41894	H30	0.39587	0.36579	0.43573
O8	0.38717	0.4973	0.81026	H31	0.41397	0.39484	0.00331
O9	0.40389	0.43808	0.38108	H32	0.31512	0.32235	0.21617
C10	0.38978	0.38968	0.23825	H33	0.33096	0.3657	-0.14858
C11	0.33428	0.36336	0.13692	H34	0.31513	0.38229	0.26715
C12	0.37273	0.65158	0.51502	O35	0.4306	0.73665	0.40039
C13	0.38719	0.70553	0.47772	H36	0.44998	0.67171	0.55209
C14	0.40913	0.63956	0.57281	H37	0.53548	0.40543	0.23968
N15	0.6125	0.41783	0.24649	H38	0.53176	0.59047	0.84769
N16	0.57557	0.43162	0.30964	H39	0.59907	0.61152	1.15914
C17	0.58776	0.47723	0.4576	H40	0.6187	0.64061	0.7277
C18	0.54804	0.49174	0.52661	H41	0.70439	0.65838	0.80881
C19	0.55986	0.53625	0.67811	H42	0.68712	0.61459	1.1712
C20	0.52041	0.55202	0.73073	H43	0.68717	0.67447	1.2204
O21	0.63186	0.50638	0.53155	O44	0.56846	0.26942	-0.09103
O22	0.61025	0.56822	0.78721	H45	0.3611	0.56158	0.76301
C23	0.62401	0.61661	0.92861	H46	0.65319	0.44572	0.28545

Supplementary Table 4. Fractional atomic coordinates for the eclipsed AA-stacking unit cell of COF-4.

Space group *P*3, $a = b = 30.81$ Å, $c = 4.06$ Å, $\alpha = \beta = 90°$, and $\gamma = 120°$<br>Pawley refinement $R_p = 2.15\%$, $R_{wp} = 2.72\%$							
Atom	x (Å)	y (Å)	z (Å)	Atom	x (Å)	y (Å)	z (Å)
C1	0.59363	0.36555	0.5	C24	0.666	0.66645	0.5
N2	0.39337	0.58739	0.5	C25	0.63132	0.34956	0.5
N3	0.43027	0.57335	0.5	C26	0.61527	0.29819	0.5
C4	0.41697	0.52159	0.5	H27	0.55211	0.33549	0.5
C5	0.45735	0.50823	0.5	H28	0.47069	0.60263	0.5
C6	0.44751	0.46021	0.5	H29	0.48479	0.424	0.23839
C7	0.48964	0.44823	0.5	H30	0.48667	0.42545	0.76646
O8	0.37263	0.48923	0.5	H31	0.40783	0.36563	0.76649
O9	0.39714	0.42087	0.5	H32	0.40785	0.36564	0.23354
C10	0.38979	0.37128	0.5	H33	0.32738	0.29632	0.61521
C11	0.334	0.33355	0.5	H34	0.31891	0.32813	0.19814
C12	0.36868	0.65044	0.5	H35	0.31368	0.34777	0.68665
C13	0.38473	0.70181	0.5	H36	0.42665	0.73063	0.5
C14	0.40637	0.63445	0.5	H37	0.44789	0.66451	0.5
N15	0.60663	0.41261	0.5	H38	0.52931	0.39737	0.5
N16	0.56973	0.42665	0.5	H39	0.51521	0.576	0.23839
C17	0.58303	0.47841	0.5	H40	0.51333	0.57455	0.76646
C18	0.54265	0.49177	0.5	H41	0.59217	0.63437	0.76649
C19	0.55249	0.53979	0.5	H42	0.59215	0.63436	0.23354
C20	0.51036	0.55177	0.5	H43	0.67893	0.67999	0.19814
O21	0.62737	0.51077	0.5	H44	0.68741	0.64817	0.61521
O22	0.60286	0.57913	0.5	H45	0.6737	0.69962	0.68665
C23	0.61021	0.62872	0.5	H46	0.57335	0.26937	0.5

Supplementary Materials characterization

Fourier transform infrared (FT-IR) spectroscopy

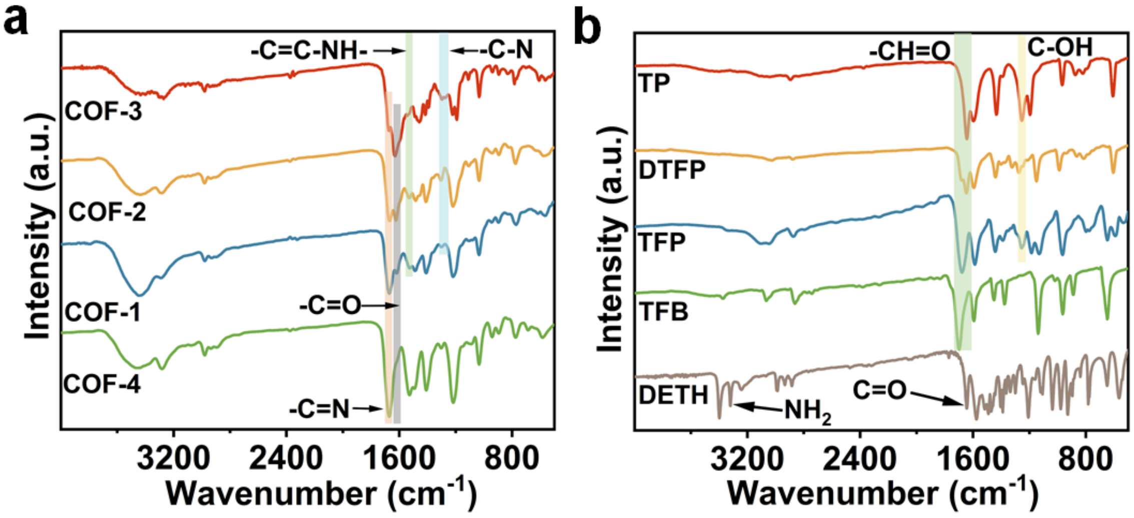

Supplementary Fig. 1 | (a) FT-IR transmittance spectra for COF-1, COF-2, COF-3, COF-4. (b) FT-IR transmittance spectra for linkers.

Crystals structures

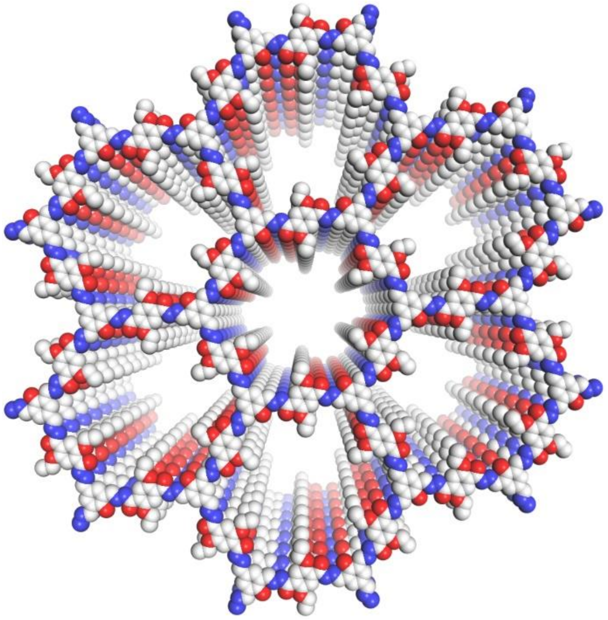

Supplementary Fig. 2 | 3D structural model of COF-1.

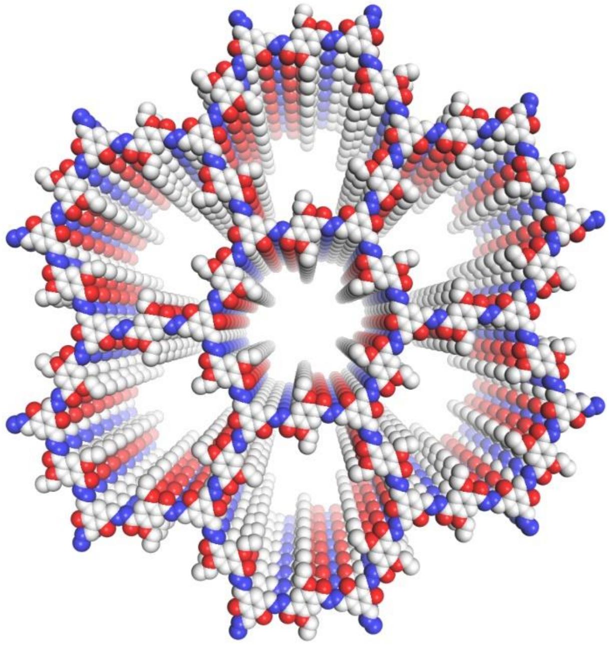

Supplementary Fig. 3 | 3D structural model of COF-2.

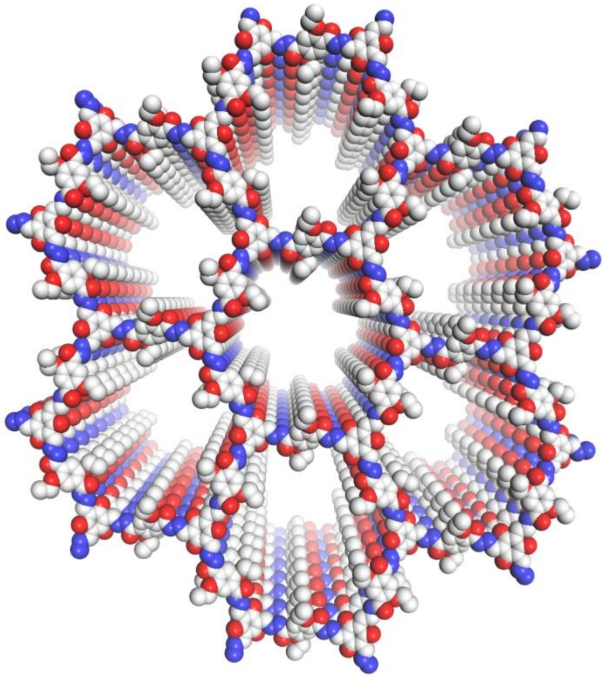

Supplementary Fig. 4 | 3D structural model of COF-3.

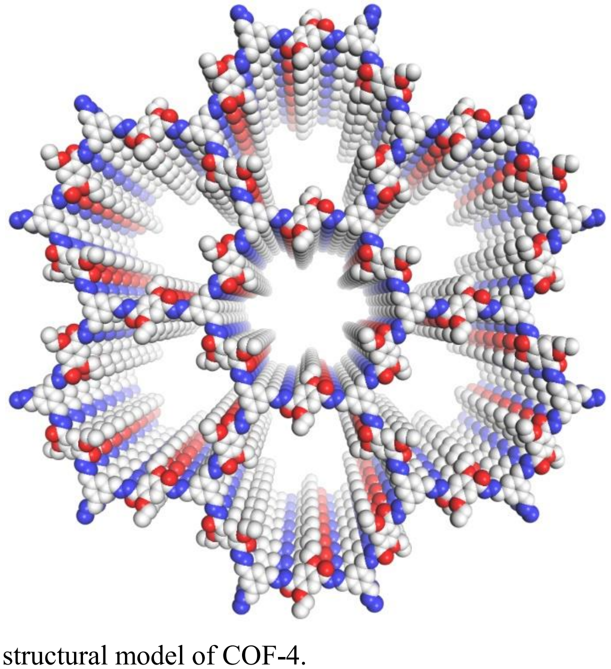

Supplementary Fig. 5 | 3D structural model of COF-4.

Thermogravimetric analysis (TGA)

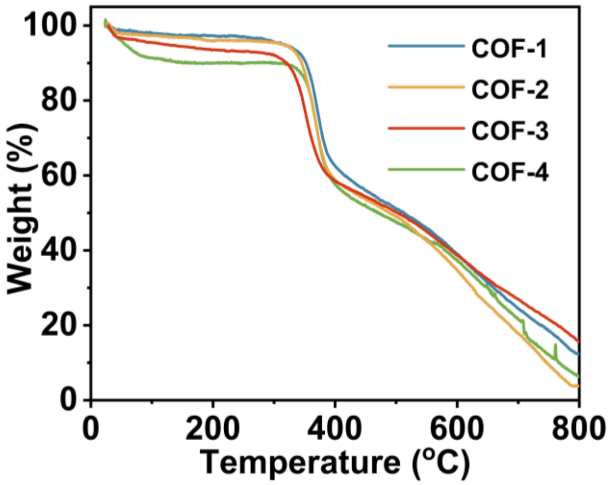

Supplementary Fig. 6 | TGA curves for COF-1, COF-2, COF-3, and COF-4. Samples were heated from room temperature to 800 °C in N_2 at 10 °C min^{-1}.

Stability tests

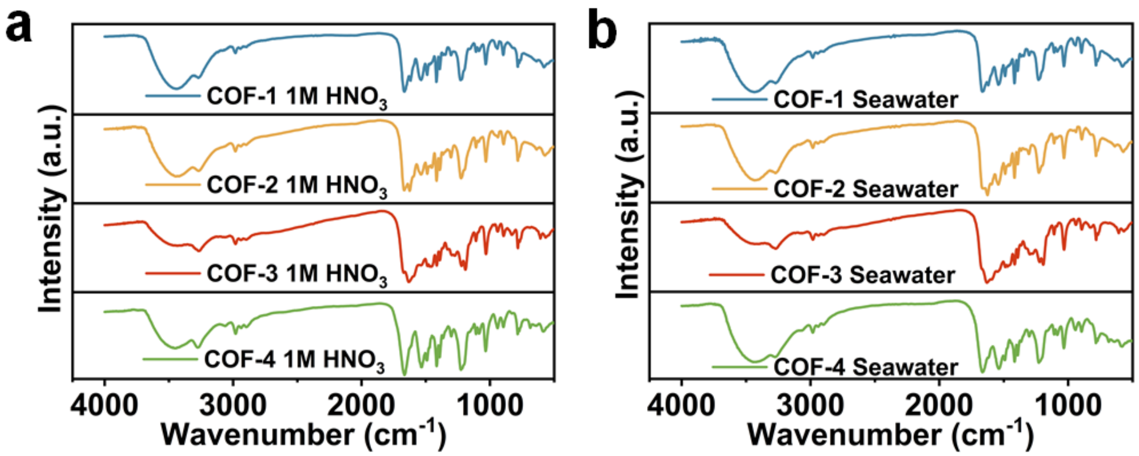

Supplementary Fig. 7 | (a) FT-IR spectra of COF-1, COF-2, COF-3, and COF-4 after treatment in 1 M HNO_3 over 24 h. (b) FT-IR spectra of COF-1, COF-2, COF-3, and COF-4 after treatment in natural seawater over 24 h.

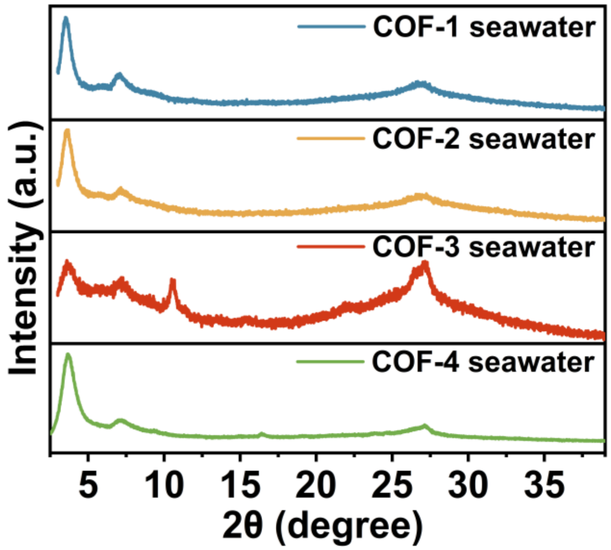

Supplementary Fig. 8 | PXRD patterns of COF-1, COF-2, COF-3, and COF-4 after treatments in natural seawater over 24 h.

Ultraviolet/visible (UV-Vis) diffuse reflectance spectroscopy

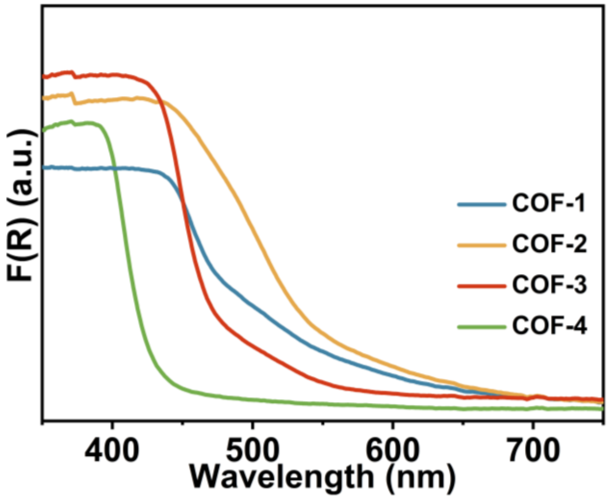

Supplementary Fig. 9 | UV-Vis diffuse reflectance spectra for COF-1, COF-2, COF-3, and COF-4.

Supplementary Methods

Photocatalytic uranium extraction and mechanism studies

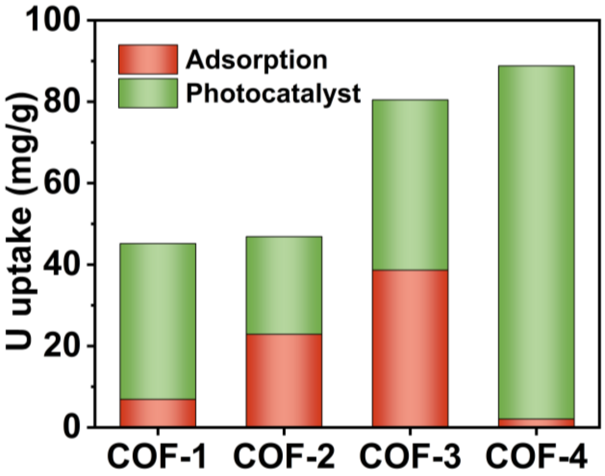

Supplementary Fig. 10 | Uranium extraction from spiked groundwater with initial uranium concentrations of ~10 ppm, using COF-1, COF-2, COF-3, and COF-4 as photocatalysts (highlighting the differences in the adsorption and photocatalytic properties of the four COFs).

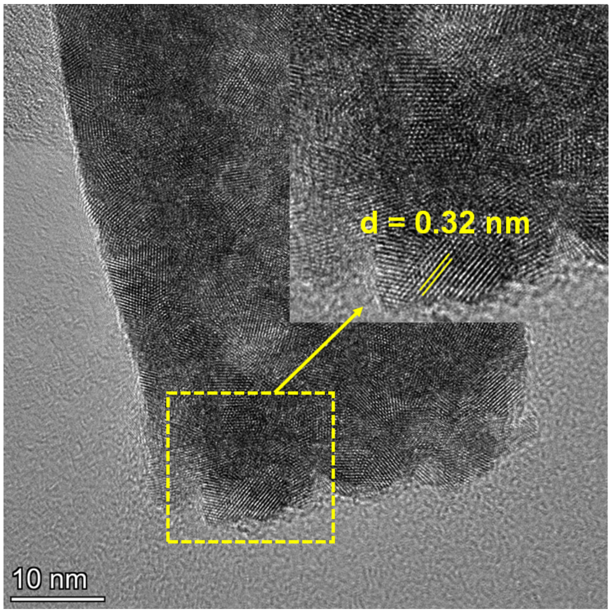

Supplementary Fig. 11 | HRTEM image of COF-4 (attached a solid nanoparticle) after photocatalysis in uranium-spiked groundwater.

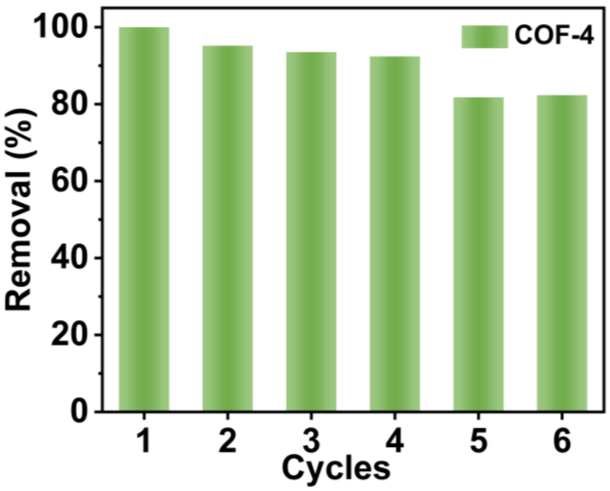

Supplementary Fig. 12 | Recycle test for uranium extraction by COF-4.

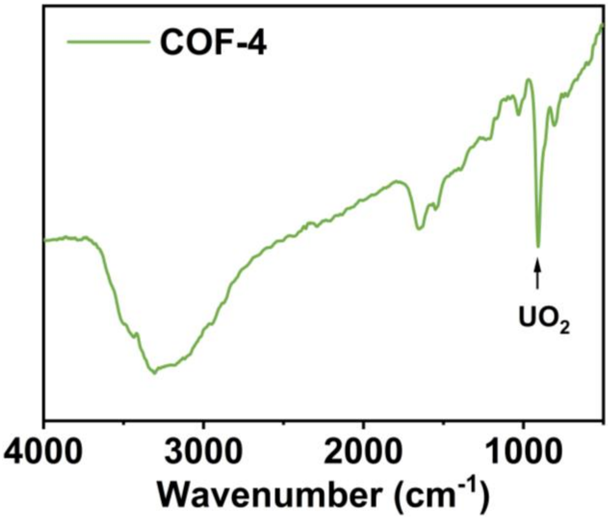

Supplementary Fig. 13 | FT-IR spectrum of COF-4 after the recycling test (i.e. after 6 cycles of use).

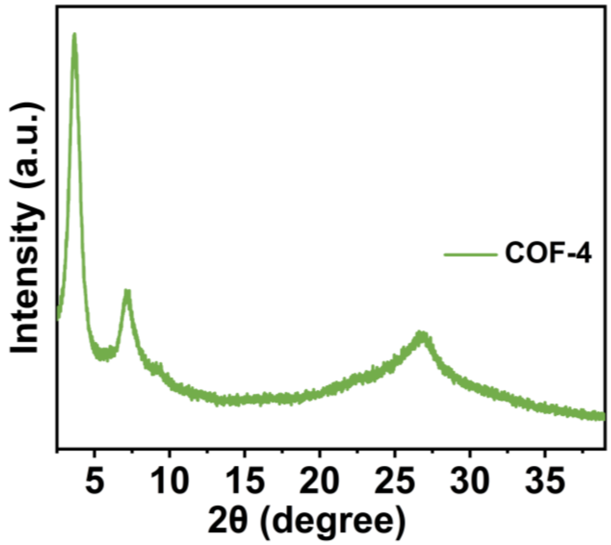

Supplementary Fig. 14 | PXRD of COF-4 after the recycling test (i.e. after 6 cycles of use).

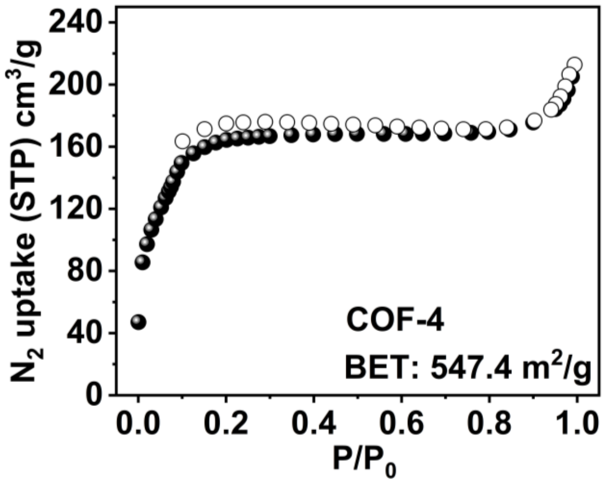

Supplementary Fig. 15 | N_2 adsorption (filled symbols) and desorption (open symbols) isotherms measured at 77 K for COF-4 after photocatalysis.

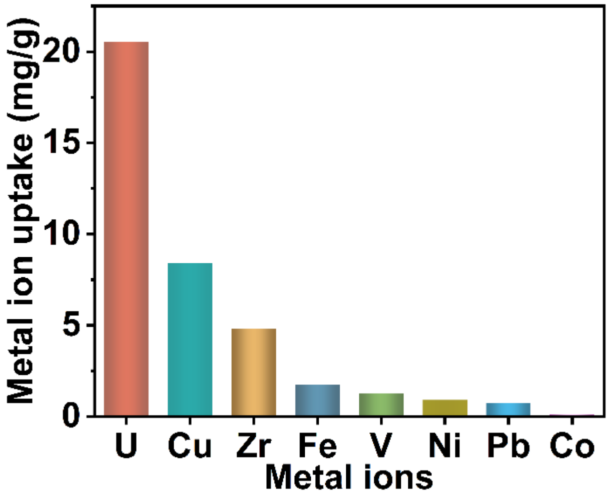

Supplementary Fig. 16 | Selectivity of COF-4 for different metals in natural seawater.

X-ray absorption spectroscopy

U L_{III}-edge X-ray absorption spectra were collected on the X-ray Absorption Spectroscopy beamline of the Shanghai Synchrotron Radiation Facility. The X-ray beam was monochromatized using a Si (111) monochromator. Fluorescence spectra were collected in transmittance mode. Incident and transmitted X-rays were monitored using gas ionization chambers. The energy scales were calibrated using a Zr foil. The U L_{III}-edge absorption spectra were processed with the Athena and Artemis programs of the IFEFFIT package.[1]

Supplementary Table 5. Summary of U L_{III}-edge EXAFS curve fitting parameters for COF-4 and COF-3 after photocatalysis.

Samples	Path	*R* (Å)	*CN*	σ^2 (Å^2)	ΔE (eV)	*R*-factor
COF-4	$U\text{-}O_{ax}$	1.81546	2.576	0.00720	8.583	0.0208
	$U\text{-}O_{eq1}$	2.16715	1.288	0.00838	8.583	0.0208
	$U\text{-}O_{eq2}$	2.39652	2.576	0.00670	8.583	0.0208
COF-3	$U\text{-}O_{ax}$	1.81501	2.8	0.00671	7.064	0.008
	$U\text{-}O_{eq1}$	2.12414	1.4	0.00170	7.064	0.008
	$U\text{-}O_{eq2}$	2.32059	2.8	0.00045	7.064	0.008

R, distance between absorber and backscattering atoms; *CN*, coordination number; σ^2, Debye-Waller factor to account for both thermal and structural disorders; ΔE, inner potential correction; *R-factor*, indicates the goodness of the fit. $U\text{-}O_{ax}$ and $U\text{-}O_{eq}$ represent axial coordination and in-plane coordination, respectively.

Supplementary Table 6. Comparison of the performance of different adsorbents for uranium extraction from natural seawater.

Material	Conditions	Capacity for U (mg/g)	Time (d)	Capacity for U (mg/g/day)	Ref.
blank membrane without Q-CS	36 mg sample	6.4	25	0.256	2
AUPM	36 mg sample	8.78	25	0.351	2
Zn^{2+}-PAO	36 mg sample	9.23	28	0.330	3
H-ABP	10 mg sample	11.5	90	0.128	4
BP-PAO	10 mg sample	11.76	56	0.21	5
DSUP fibers	10 mg sample	17.45	3	5.82	6
AO-PIM-1	10 mg sample	9.03	28	0.323	7
UiO-66-3C4N	20 mg sample	6.85	28	0.245	8
DNA-UEH	10 mg sample	6.06	6	1.01	9
PAO/Alg NFs	120 mg sample	8.42	56	0.151	10
PPH-OP	10 mg sample	7.12	21	0.339	11
PAF-CS	5 mg sample	6	21	0.286	12
POP_1-AO	2.5 mg sample	8.4	56	0.15	13
SSUP fiber	10 mg sample	12.33	3.5	3.523	14
AO-HNTs	10 mg sample	9.01	30	0.300	15
MS@PIDO/Alg sponge		5.84	56	0.104	16
COF-HHTF-AO	5 mg sample	5.12	25	0.205	17
Tp-DBD	5 mg sample	10.31	8	1.289	18
POP-oNH_2-AO	5 mg sample	4.36	56	0.078	19
p(2DVB-VBC)-2PAN	10 mg sample	1.99	27	0.074	20
NDA-TN-AO	5 mg	6.07	27	0.225	21
MIL-101-OA	100 mg sample	4.6	5	0.92	22
Anti-UiO-66	5 mg sample	4.62	30	0.154	23
PT-BN-AO	5 mg sample	5.78	27	0.214	24
Cp-1:12	10 mg sample	0.55	28	0.0196	25
Fe-N_x-C-R	6 mg sample	1.2	1	1.2	26
MUU_{re}	8 mg sample	7.35	16	0.46	27
MISS-PAF-1	5 mg sample	5.76	56	0.103	28
PPA@MISS-PAF-1		5.4/13/16.5	14/56/90	0.385/0.232/0.183	29

MIGPAF-13	4 mg sample	8.01	28	0.286	[30]
UiO-66-AO	1 mg sample	2.68	3	0.893	[31]
COF 4-Pd-AO	10 mg	13.86	3	4.62	[32]
SMON–PAO	10 mg sample	9.59	56	0.171	[33]
TFCH		15/17.9	15/30	0.596	[34]
Zr/Ti-MOF-25	100 mg sample	8.66	28	0.309	[35]
i-MZIF90(50)	4.8 mg sample	6/28.2	2/25	3.0/1.128	[36]
COF 2-Ru-AO	10 mg sample	7.36	3	2.45	[37]
Adsorbent fibers		5.22	49	0.107	[38]
COF-4	9 mg sample	20.52	3	6.84	This work

Density functional theory (DFT) calculations

The geometries of the four different COF materials were reduced to a simple repeating unit. These COF units and adsorption structures of $[U(VI)O_2(H_2O)_2]^{2+}$@COF and $[U(IV)O_2(H_2O)_2]$@COF^{2+} were all optimized with dispersion corrected density functional theory (DFT-D_3) at the PBE_0-D_3/def_2-SVP[39, 40] + SDD[41-43] level using the Gaussian 16 program. Here, the SDD effective core potential was used to describe the atomic orbital and relativistic effect of heavy element uranium. In our photocatalytic experiments, the COFs played the role of U(VI) reductants, therefore the electron numbers of these complex structures were conserved, with the main difference between $[U(VI)O_2(H_2O)_2]^{2+}$@COF and $[U(IV)O_2(H_2O)_2]$@COF^{2+} being that the former was in the singlet state while the later was in triplet state. Vibrational frequency analyses were carried out for these optimized structures with the same calculation method to obtain the zero-point energy and free energy corrections. In order to obtain the electron energy with high accuracy (which has the major impact on the accuracy of Gibbs free energy), single point calculations for these optimized structures with PBE0-D_3 functional and def2TZVP+SDD basis set were performed. Finally, the single point energy was added to the free energy correction calculated before to obtain the Gibbs free energy. The atomic dipole moment corrected Hirshfeld (ADCH) atomic charges,[44, 45] from which we can analyze the charge transfer directly, were obtained by population analysis from the wave function file of DFT calculation using Multiwfn.[46] The adsorption free energy of the complex was calculated from the formular:

$$G(\text{adsorb}) = G(\text{A+B}) - G(\text{A}) - G(\text{B})$$

where G(A) and G(B) is the Gibbs free energy of isolated molecules, G(A+B) is the total free energy of the complex structure.

In order to investigate the photophysical properties, the excited electronic structures of the $[U(VI)O_2(H_2O)_2]^{2+}$@COF complex structures were calculated at the PBE0-D_3/SVP+SDD level with the time-dependent density functional theory (TDDFT) method, with the excited energy and oscillator strength also being obtained from the calculations. All the calculations were performed using the Gaussian 16 program. The

$[U(IV)O_2(H_2O)_2]@COF^{2+}$ product of the photochemical reactions were in the triplet state, meaning that charge transfer from COFs to $[U(VI)O_2(H_2O)_2]^{2+}$ during the electron transition involved intersystem crossing (ISC) from S_1 to T_1 excited state. The natural transition orbitals (NTOs) and the contributions of molecular orbital transitions were obtained by electron excitation analysis from the transition density matrix of TD-DFT calculation using Multiwfn program. The visualization of the natural transition orbitals used the Visual Molecular Dynamic program (VMD) .[47] From the NTOs, we can observe the spatial distributions of the hole and electron of the S_1 excited state, with the hole being located on the COF fragment whilst the electron was located at $[U(VI)O_2(H_2O)_2]^{2+}$ fragment, providing direct evidence of excited state charge transfer from the donor (COF) to acceptor ($[U(VI)O_2(H_2O)_2]^{2+}$).

There is a competitive relationship between the photophysical process of fluorescence emission from the S_1 excited state to ground state and the intersystem crossing (ISC) process from S_1 to T_1 excited state. Therefore, suppressing the radiative transition is favorable for our desired photochemical reaction. The calculations demonstrated that the $[U(VI)O_2(H_2O)_2]^{2+}$@COF-4 possessed the minimum S_1 excited state oscillator strength, possible explaining by COF-4 possessed the strongest photochemical reaction activity.

Supplementary Table 7. Summary of the bond lengths of COF photocatalysts before and after electron transfer.

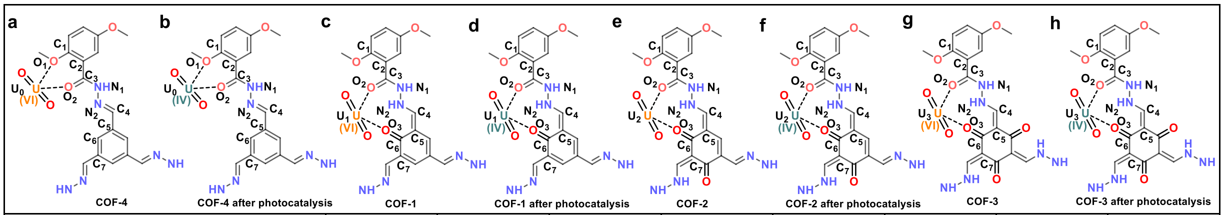

	**a**	**b**	**c**	**d**	**e**	**f**	**g**	**h**
$U(VI)_0$-O_1	2.800	3.245	-	-	-	-	-	-
$U(IV)_0$-O_2	2.300	2.522	-	-	-	-	-	-
$U(VI)_1$-O_2	-	-	2.550	2.629	-	-	-	-
$U(IV)_1$-O_3	-	-	2.335	2.640	-	-	-	-
$U(VI)_2$-O_2	-	-	-	-	2.473	2.603	-	-
$U(IV)_2$-O_3	-	-	-	-	2.334	2.602	-	-
$U(VI)_3$-O_2	-	-	-	-	-	-	2.452	2.580
$U(IV)_3$-O_3	-	-	-	-	-	-	2.355	2.533
O_1-C_1	1.254	1.233	-	-	-	-	-	-
O_2-C_3	1.403	1.420	1.237	1.230	1.246	1.235	1.286	1.266
O_3-C_6	-	-	1.302	1.252	1.295	1.262	1.412	1.412
N_1-C_3	1.414	1.440	1.413	1.413	1.412	1.412	1.348	1.363
N_2-C_4	1.340	1.342	1.472	1.471	1.472	1.476	1.473	1.475
N_1-N_2	1.471	1.493	1.364	1.374	1.353	1.361	1.355	1.346
C_1-C_2	1.348	1.347	1.342	1.336	1.347	1.347	1.318	1.307
C_2-C_3	1.281	1.280	1.295	1.292	1.305	1.303	1.397	1.411
C_4-C_5	1.460	1.461	1.434	1.440	1.412	1.415	1.441	1.422
C_5-C_6	1.406	1.407	1.442	1.466	1.439	1.433	1.430	1.461
C_6-C_7	1.395	1.396	1.424	1.462	1.431	1.472	1.247	1.235

Antibiofouling measurements

Antibiofouling measurements were carried out according to a reported literature procedure.[5] Briefly, Marine bacteria (mixture) was used as an indicator strain to determine the antibiofouling activity of COF-3 and COF-4. COF-3 or COF-4 was dispersed into Marine bacteria/fresh Luria-Bertani broth at a dispersion concentration of 0.08 mg/mL. After incubation at 37 °C for 4 h in the dark, the concentration of the marine bacteria was determined by the plate count method. Similar experiments were performed, expect the incubation step at 37 °C for 4 h was carried out under visible light irradiation. Bacterial cultures were used as a comparison. It should be noted that he marine bacterial colonies could not be isolated into pure cultures.

Inhibition rates (*IR*) were calculated according to the following equation:

$$IR = \frac{(C_{\mathrm{i}} - C_{\mathrm{a}}) \times V}{C_{\mathrm{i}}} \times 100\%$$

Where C_{i} (CFU/mL) and C_{a} (CFU/mL) are the microbial concentrations in cultures untreated and treated by COF-3 or COF-4, respectively.

Supplementary Table 8. The inhibition rates of COF-3 and COF-4 on marine bacteria.

Catalysts	Inhibition rate in dark	Inhibition rate under visible light
COF-3	39.92	85.88
COF-4	46.23	89.71

Supplementary References

1. Ravel, B. & Newville, M. ATHENA, ARTEMIS, HEPHAESTUS: data analysis for X-ray absorption spectroscopy using IFEFFIT. *J. Synchrotron Radiat.* **12**, 537-541 (2005).
2. Sun, Y., et al. Antibiofouling ultrathin poly(amidoxime) membrane for enhanced U(VI) recovery from wastewater and seawater. *ACS. Appl. Mater. Interfaces* **13**, 21272-21285 (2021).
3. Yan, B., Ma, C., Gao, J., Yuan, Y. & Wang, N. An ion-crosslinked supramolecular hydrogel for ultrahigh and fast uranium recovery from seawater. *Adv. Mater*. **32**, 1906615 (2020).
4. Xu, X., et al. 3D hierarchical porous amidoxime fibers speed up uranium extraction from seawater. *Energy Environ. Sci.* **12**, 1979-1988 (2019).
5. Yuan, Y. H., et al. Photoinduced multiple effects to enhance uranium extraction from natural seawater by black phosphorus nanosheets. *Angew. Chem. Int. Ed.* **59**, 1220-1227 (2019).
6. Yu, Q., et al. Spidroin-inspired, high-strength, loofah-shaped protein fiber for capturing uranium from seawater. *Angew. Chem. Int. Ed.* **132**, 16131 (2020).
7. Yang, L., et al. Bioinspired hierarchical porous membrane for efficient uranium extraction from seawater. *Nat. Sustain.* **5***,* 71-80 (2021).
8. Yuan, Y., et al. A bio-inspired nano-pocket spatial structure for targeting uranyl capture. *Angew. Chem. Int. Ed.* **59**, 4262-4268 (2020).
9. Yuan, Y., et al. DNA nano-pocket for ultra-selective uranyl extraction from seawater. *Nat. Commun*. **11**, 5708 (2020).
10. Xu, X., et al. Aqueous solution blow spinning of seawater-stable polyamidoxime nanofibers from water-soluble precursor for uranium extraction from seawater. *Small Methods* **4**, 2000558 (2020).
11. Yuan, Y., et al. Selective extraction of uranium from seawater with biofouling-resistant polymeric peptide. *Nat. Sustain.* **4**, 708 (2021).
12. Li, Z., et al. Constructing amidoxime-modified porous adsorbents with open architecture for cost-effective and efficient uranium extraction. *Chem. Sci.* **11**, 4747-4752 (2020).
13. Song, Y., et al. Nanospace decoration with uranyl-specific "hooks" for selective uranium extraction from seawater with ultrahigh enrichment index. *ACS Cent. Sci.* **7**, 1650-1656 (2021).
14. Yuan, Y., et al. Ultrafast and highly selective uranium extraction from seawater by hydrogel-like spidroin-based protein fiber. *Angew. Chem. Int. Ed.* **58**, 11785-11790 (2019).
15. Zhao, S., et al. A dual-surface amidoximated halloysite nanotube for high-efficiency economical uranium extraction from seawater. *Angew. Chem. Int. Ed.* **58**, 14979-14985 (2019).
16. Wang, D., et al. A marine-inspired hybrid sponge for highly efficient uranium extraction from seawater. *Adv. Funct. Mater.* **29**, 1901009 (2019).

17. Cheng, G., et al. Extremely stable amidoxime functionalized covalent organic frameworks for uranium extraction from seawater with high efficiency and selectivity. *Sci. Bull.* **66**, 1994-2001 (2021).
18. Cui, W. R., et al. Low band gap benzoxazole-linked covalent organic frameworks for photo-enhanced targeted uranium recovery. *Small* **17**, 2006882 (2021).
19. Sun, Q., et al. Bio-inspired nano-traps for uranium extraction from seawater and recovery from nuclear waste. *Nat. Commun*. **9**, 1644 (2018).
20. Yue, Y., et al. Seawater uranium sorbents: preparation from a mesoporous copolymer initiator by atom-transfer radical polymerization. *Angew. Chem. Int. Ed.* **52**, 13458-13462 (2013).
21. Cui, W. R., et al. Regenerable covalent organic frameworks for photo-enhanced uranium adsorption from seawater. *Angew. Chem. Int. Ed.* **59**, 17684 (2020).
22. Wu, H., et al. Control of pore chemistry in metal-organic frameworks for selective uranium extraction from seawater. *Micropor. Mesopor. Mater.* **288**, 109567 (2019).
23. Yu, Q., et al. A Universally applicable strategy for construction of anti-biofouling adsorbents for enhanced uranium recovery from seawater. *Adv. Sci.* **6**, 1900002 (2019).
24. Cui, W.-R., et al. High-efficiency photoenhanced extraction of uranium from natural seawater by olefin-linked covalent organic frameworks. *ACS ES&T Water*, **1**, 440-448 (2020).
25. Yue, Y., et al. Polymer-coated nanoporous carbons for trace seawater uranium adsorption. *Sci. China Chem.* **56**, 1510-1515 (2013).
26. Yang, H., et al. Functionalized iron-nitrogen-carbon electrocatalyst provides a reversible electron transfer platform for efficient uranium extraction from seawater. *Adv. Mater.* 33, 2106621 (2021).
27. Feng, L., et al. In-situ synthesis of uranyl-imprinted nanocage for selective uranium recovery from seawater. *Angew. Chem. Int. Ed.* **61**, 202101015 (2021).
28. Yuan, Y., et al. A molecular coordination template strategy for designing selective porous aromatic framework materials for uranyl capture. *ACS Cent. Sci*. **5**, 1432-1439 (2019).
29. Wang, Z., et al. Constructing an ion pathway for uranium extraction from seawater. *Chem* **6**, 1683-1691 (2020).
30. Wang, Z., et al. Constructing uranyl-specific nanofluidic channels for unipolar ionic transport to realize ultrafast uranium extraction. *J. Am. Chem. Soc.* **143**, 14523-14529 (2021).
31. Chen, L.,et al. Ultrafast and efficient extraction of uranium from seawater using an amidoxime appended metal-organic framework. *ACS. Appl. Mater. Interfaces* **9**, 32446-32451 (2017).
32. Hao, M., et al. Converging cooperative functions into the nanospace of covalent organic frameworks for efficient uranium extraction from seawater. *CCS Chem.* **4**, 2294-2307 (2022).
33. Yuan, Y., et al. Rational design of porous nanofiber adsorbent by blow-spinning with ultrahigh uranium

recovery capacity from seawater. *Adv. Funct. Mater.* **29**, 1805380 (2019).

34. Kaushik, A., et al. Large-area self-standing thin film of porous hydrogen-bonded organic framework for efficient uranium extraction from seawater. *Chem* **8**, 2749-2765 (2022).

35. Liu, T., et al. Defect-engineered metal-organic framework with enhanced photoreduction activity toward uranium extraction from seawater. *Cell Rep. Phys. Sci.* **3**, 100892 (2022).

36. Mollick, S., et al. Benchmark uranium extraction from seawater using an ionic macroporous metal–organic framework. *Energy Environ. Sci.* **15**, 3462-3469 (2022).

37. Hao, M., et al. Modulating uranium extraction performance of multivariate covalent organic frameworks through donor–acceptor linkers and amidoxime nanotraps. *JACS Au* **3**, 239-251 (2023).

38. Brown, S., et al. Uranium adsorbent fibers prepared by atom-transfer radical polymerization (ATRP) from poly(vinyl chloride)-co-chlorinated poly(vinyl chloride) (PVC-co-CPVC) fiber. *Ind. Eng. Chem. Res.* **55**, 4139-4148 (2016).

39. Adamo, C. & Barone, V. Toward reliable density functional methods without adjustable parameters: The PBE0 model. *J. Chem. Phys.* **110**, 6158-6170 (1999).

40. Weigend, F. & Ahlrichs, R. Balanced basis sets of split valence, triple zeta valence and quadruple zeta valence quality for H to Rn: Design and assessment of accuracy. *Phys. Chem. Chem. Phys.* **7**, 3297-3305 (2005).

41. Cao, X., Dolg, M. & Stoll, H. Valence basis sets for relativistic energy-consistent small-core actinide pseudopotentials. *J. Chem. Phys.* **118**, 487-496 (2003).

42. Cao, X. & Dolg, M. Segmented contraction scheme for small-core actinide pseudopotential basis sets. *J. Mol. Struct. Theochem* **673**, 203-209 (2004).

43. Küchle, W., Dolg, M., Stoll, H. & Preuss, H. Energy-adjusted pseudopotentials for the actinides. Parameter sets and test calculations for thorium and thorium monoxide. *J. Chem. Phys.* **100**, 7535-7542 (1994).

44. Hirshfeld, F. L. Bonded-atom fragments for describing molecular charge densities. *Theor. Chim. Acta* **44**, 129-138 (1977).

45. Lu, T. & Chen, F. Atomic dipole moment corrected Hirshfeld population method. *J.Theor. Comput. Chem.* **11**, 163-183 (2012).

46. Lu, T. & Chen, F. Multiwfn: a multifunctional wavefunction analyzer. *J. Comput. Chem.* **33**, 580-592 (2012).

47. Humphrey, W., Dalke, A. & Schulten, K. VMD: visual molecular dynamics. *J. Mol. Graph.* **14**, 33-38, 27-38 (1996).
